# Evaluation of short-term intraocular pressure changes after intravitreal injection of Conbercept in patients with diabetic macular edema

**DOI:** 10.3389/fphar.2022.1025205

**Published:** 2022-12-12

**Authors:** Yunyan Hu, Yunkao Zeng, Jing Yang, Xiaomin Zeng, Dan Cao, Biqun Ou, Guanrong Zhang, Liang Zhang

**Affiliations:** ^1^ Department of Ophthalmology, Guangdong Provincial People’s Hospital, Guangdong Academy of Medical Sciences, Guangzhou, China; ^2^ State Key Laboratory of Ophthalmology, Zhongshan Ophthalmic Center, Sun Yat-sen University, Guangzhou, China; ^3^ Statistics Section, Guangdong Provincial People’s Hospital, Guangdong Academy of Medical Sciences, Guangzhou, China; ^4^ School of Medicine, South China University of Technology, Guangzhou, China

**Keywords:** intraocular pressure, risk factor, anti-VEGF, Conbercept, diabetic macular edema

## Abstract

**Background:** The study concerning the influence of Conbercept, which is an anti-Vascular endothelial growth factor (VEGF) agent, in intraocular pressure (IOP) spike is limited and warrants further investigation. The current study aimed to investigate the changes of intraocular pressure after intravitreal injection (IVI) of Conbercept and evaluate the risk factors associated with intraocular pressure spikes.

**Methods:** Patients with diabetic macular edema receiving intravitreal injection of 0.05 ml (0.5 mg) Conbercept were involved in the study. All patients underwent slit lamp examination to determine the status of phakia/pseudophakia. The axial length was measured using IOL Master 500 before intravitreal injection. Patients underwent a Conbercept intravitreal injection with a 30-gauge needle in a standard fashion. The intraocular pressure was measured 2 min before injection, and 2, 10, 30 min, 1, 2, 5, 24 h after injection using a rebound tonometer. The changes of intraocular pressure and the relevant risk factors were evaluated. Patients were subdivided into phakic group and pseudophakic group to analyze the effect of lens status on intraocular pressure changes.

**Results:** Forty patients with a mean age of 62.48 ± 12.22 years were included in the study. The mean intraocular pressure values at baseline and 2, 10, 30 min, 1, 2, 5, 24 h after injection were 14.81 ± 3.13 mmHg, 26.80 ± 9.43 mmHg, 18.76 ± 6.16 mmHg, 16.54 ± 5.94 mmHg, 15.64 ± 3.75 mmHg, 14.46 ± 3.03 mmHg, 14.10 ± 1.88 mmHg, 14.23 ± 2.71 mmHg respectively. The intraocular pressure after injection for 2, 10 min was significantly higher than baseline (*p* < 0.001, *p* = 0.001, respectively). The intraocular pressure between baseline and post-injection for 30 min or beyond were comparable (all *p* > 0.05). No significant difference was found between the phakic group and pseudophakic group (*p* = 0.422). The changes of intraocular pressure were positively correlated with age (*r* = 0.329, *p* = 0.038), but negatively with axial length (*r* = −0.472, *p* = 0.002).

**Conclusion:** intravitreal injection of Conbercept may cause rapid spike of intraocular pressure, but is safe with respect to short-term changes. The intraocular pressure in patients with older age and shorter axial length is more likely to be higher after intravitreal injection.

## 1 Introduction

Diabetic macular edema (DME) is characterized by accumulation of exudative fluid at the macula and remains a major cause of visual impairment in patients with diabetic retinopathy (Tan et al., 2017). Vascular endothelial growth factor (VEGF) is the essential molecular mediator in the pathogenesis of DME (Das et al., 2015). In order to combat the increased level of ocular VEGF and inhibit the pathologic effect, anti-VEGF agents like ranibizumab, aflibercept, bevacizumab have been used in patients with DME. To date, both randomized clinical trials and real-world data have conclusively proved that anti-VEGF agents are effective in the treatment of DME (Heier et al., 2016; Wells et al., 2016). Thus, Intravitreal injection (IVI) of anti-VEGF agents has been widely used in managing DME.

Despite the therapeutic advances of the anti-VEGF agents, repeated administrations are needed to achieve good anatomical and functional improvements. The accompanying short-term and long-term complications of repeated IVI like elevated IOP have been arousing our concern. IVI leads to sudden increase of volume in the vitreous cavity and subsequently increase the IOP. The course of IOP changes needs to be closely monitored and the safety outcome should be evaluated. Previous studies have found that the increased IOP immediately following IVI was volume-driven and generally transient and well tolerated (Mojica et al., 2008; Abedi et al., 2013). However, those pooled studies mainly focused on ranibizumab, aflibercept and bevacizumab (El Chehab et al., 2016; Kato et al., 2019/05; Fuest et al., 2014). Conbercept (KH902; Chengdu Kanghong Biotech Co., Ltd., Sichuan, China) is a recombinant fusion protein designed as a receptor decoy with high affinity for placental growth factor and all VEGF isoforms (Wang et al., 2013). The study concerning the influence of Conbercept in IOP spike is limited and warrants further investigation.

Non-contact tonometer (NCT) was used in the majority of previous study focusing on the current issue. However, NCT is not that reliable in patients with elevated IOP, because comparative studies have shown lower correlations with Goldmann applanation tonometer (GAT) in higher IOP ranges (Shields, 1980). Study also found that NCT showed greater overestimate of IOP in moderate, higher IOP subjects and showed a lower agreement with GAT compared with iCare Pro rebound tonometer (Icare, Tiolat Oy, Helsinki, Finland) (Chen et al., 2019). Thus, IOP measurements determined by iCare Pro rebound tonometer maybe more suitable in subjects with increased IOP after IVI. Besides, iCare PRO is a portable device using a disposable and clean probe, which ensures a better and in-time monitoring of IOP in sterile situation. The current study aimed to evaluate the short-term IOP changes in patients receiving Conbercept injections and investigate the associated factors affecting the changes.

## 2 Materials and methods

### 2.1 Subjects

This prospective observational study included consecutive patients receiving IVI of Conbercept at Guangdong Provincial People’s Hospital, Guangzhou, China, from October 2018 to June 2019. The study abided by the principles of the Declaration of Helsinki and was approved by the Research Ethics Committee of the Guangdong Provincial People’s Hospital (ethical approval number: 2018233H). Informed consent was obtained from every subject before enrollment. We included DME patients who were older than 18-year old and able to understand and sign the consent form, and the initial IOP was less than 21 mmHg. Exclusion criteria were any kind of glaucoma or suspected glaucoma (IOP > 21 mmHg and/or cup to disc ratio >0.5), a history of intraocular surgery during the past 3 months, current use of steroid eye drops or IOP-lowering drops and any ocular surface disease precluding a reliable IOP measurement.

### 2.2 Ophthalmologic examinations and intravitreal injection

All subjects underwent a complete ophthalmologic examination before IVI, including best-corrected VA (BCVA), slit-lamp examination, axial length measurement and IOP evaluation. The slit lamp exam was used to determine the status of phakia/pseudophakia and cornea. Axial length was measured with IOL Master 500 optical biometer (Carl Zeiss Meditec, Jena, Germany). ICare Pro was used to measure the IOP. It is a portable device based on the induction of rebound principle and the detailed information was reported by previous studies (Borrego Sanz et al., 2016; Nakakura, 2018). In a single measurement, IOP was detected for 6 times and the result was provided as a mean value automatically by the device after excluding the lowest and highest readings. In total, three mean IOP measurements were obtained and averaged. All the measurements of IOP were examined by the same trained ophthalmologist (Y.K.Z). The first IOP was measured 2 min before injection in a sitting position. After that, patients received IVI of 0.05 ml (0.5 mg) Conbercept with a 30-gauge needle in a standard fashion by the same experienced ophthalmologist (Y.Y.H) (Gismondi et al., 2009). For pseudophakia, the injection site was 3.5 mm posterior to the limbus, while for phakia it was 4 mm posterior to the limbus. Immediately after the injection, the needle was removed carefully and a sterile cotton applicator was used to prevent reflux. Subsequently, the IOP was measured with a new probe at 2, 10, 30 min, 1, 2, 5, 24 h after injection in sitting position. Antibiotic eyedrops were applied at the end of the procedure and administered for 7 days.

### 2.3 Statistical analysis

Data analyses were performed using SPSS software Version 26.0 (SPSS Inc., Chicago, IL). Demographic and outcome data were summarized by frequency for categorical variables and mean ± standard deviation (SD) for continuous variables. Normality of continuous variables was assessed by the Kolmogorov-Smirnov test. The changes of IOP were defined as the IOP after IVI minus the baseline IOP. To estimate mean changes in IOP, a linear mixed model for repeated measures was used, taking into account the correlations between individuals’ repeated measures over time. Least-squares means with 95% confidence intervals (CI) were reported, adjusting for gender, age, axial length and lens status. Furthermore, we conducted subgroup analyses for the changes in IOP after stratification by lens status. Pearson correlation coefficient was used to evaluate the linear correlation between the IOP changes in the initial 2 min and clinical parameters. A *p*-value less than 0.05 was considered as statistically significant.

## 3 Results

Forty eyes of 40 patients with DME were included in the study. The mean age was 62.48 ± 12.22 years (range: 39–88). There were 26 male and 14 female with a mean axial length of 23.20 ± 1.06 mm (range: 21.33–25.21 mm). Among them, 57.5% (23/40) patients were phakic and 42.5% (17/40) were pseudophakic. The patients were subdivided into phakic group and pseudophakic group according to the status of lens. The basic clinical characteristics and demographics of the patients were shown in Table 1.

**TABLE 1:** Clinical characteristics and demographics of the subjects.

Variables	All subjects	Phakic group	Pseudophakic group	*p*
Number	40	23	17	N/A
Age, years	62.48 ± 12.22	59.35 ± 13.94	66.71 ± 13.88	0.106*
Gender (male/female)	26/14	17/6	9/8	0.169*
Laterality (OD/OS)	19/21	13/10	8/9	0.554*
Axial length (mm)	23.20 ± 1.06	23.09 ± 1.04	23.35 ± 1.10	0.441*

OS, Oculus sinister (left eye); OD, Oculus dexter (right eye).

*Compared between phakic group and pseudophakic group.

**FIGURE 1:**
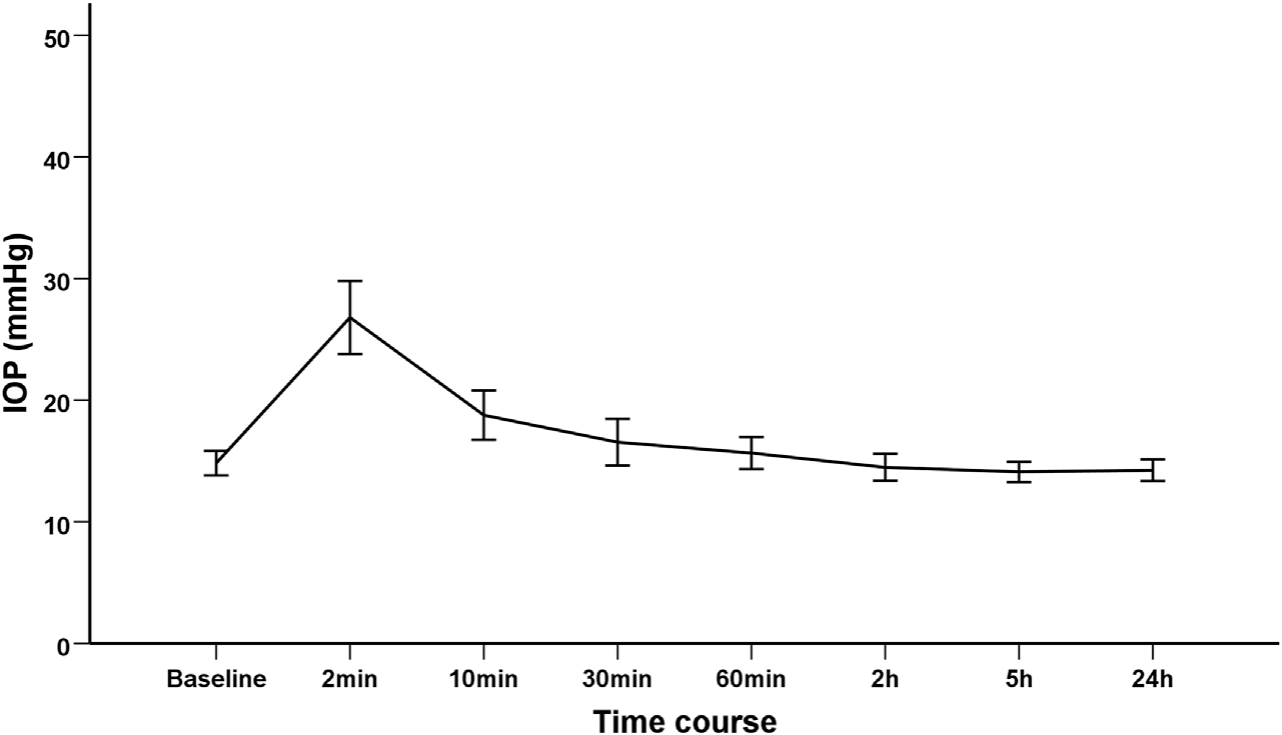
The trend of intraocular pressure changes after intravitreal injection of Conbercept in diabetic macular edema patients.

Dynamic changes in IOP were demonstrated in Table 2. The mean IOP was 14.81 ± 3.13 mmHg at baseline, which dramatically increased to 26.80 ± 9.43 mmHg at 2 min after IVI. Thereafter, the IOP declined from 10 min after IVI, and progressively returned to its respective initial level (Figure 1). Relative to the baseline value, the mean IOP significantly increased by 11.99 mmHg (95% CI 9.15–14.83) and 3.95 mmHg (95% CI 1.72–6.19) at 2 and 10 min after IVI, respectively (both *p* < 0.01). Since 30 min after IVI, no significant difference was observed in IOPs between each subsequent follow-up and the baseline value. The results remained consistent even after adjusting for gender, age, axial length and lens status (Table 2). Only three patients had an IOP of 25 mmHg or higher at 30 min. The IOP spikes in all patients resolved without medication or any surgical intervention. None of those eyes in the current study required anterior chamber paracentesis. No ocular adverse event was found during the study follow-up period.

**TABLE 2:** Changes of intraocular pressure after intravitreal injection and comparison between each time point.

	IOP (mean ± SD,mmHg)	Change from baseline (95% CI)	*p*	Change from baseline (95% CI)*	*p*
Baseline	14.81 ± 3.13		—		—
2 min	26.80 ± 9.43	11.99 (9.15–14.83)	<0.001	11.90 (8.64–15.15)	<0.001
10 min	18.76 ± 6.16	3.95 (1.72–6.19)	0.001	3.91 (1.68–6.14)	0.001
30 min	16.54 ± 5.94	1.73 (−0.42–3.87)	0.113	2.03 (−0.19–4.25)	0.072
60 min	15.64 ± 3.75	0.83 (−0.81–2.47)	0.314	0.79 (−0.80–2.38)	0.323
2 h	14.46 ± 3.03	−0.35 (−1.81–1.12)	0.64	−0.33 (−1.79–1.14)	0.656
5 h	14.10 ± 1.88	−0.71 (−1.99–0.56)	0.267	−0.73 (−1.98–0.52)	0.248
24 h	14.23 ± 2.71	−0.58 (−1.90–0.74)	0.381	−0.75 (−1.99–0.50)	0.237

*Adjusted for gender, age, axial length and status of lens. The *β* value (95% CI) and *p*-value of these covariates are listed as follow: gender −0.22 (−1.11–0.68), *p* = 0.632; age −0.02 (−0.05–0.01), *p* = 0.116; axial length −0.08 (−0.50–0.34), *p* = 0.707; lens status 1.06 (0.22–1.91), *p* = 0.014. IVI, intravitreal injection; CI, confidence interval; IOP, intraocular pressure.

**FIGURE 2:**
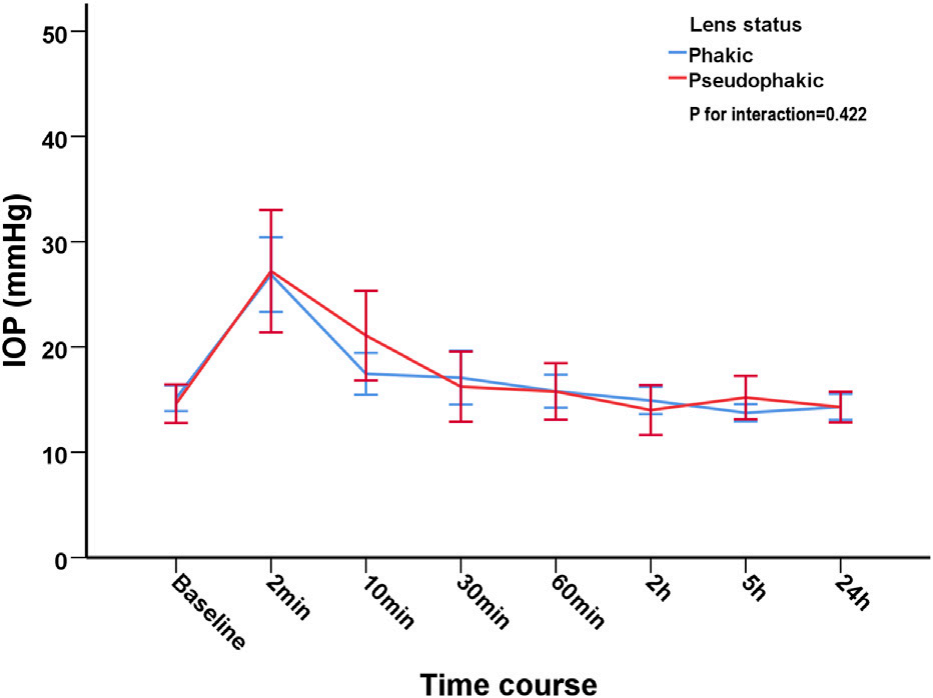
The trend of intraocular pressure changes after intravitreal injection of Conbercept in phakic group and pseudophakic group.

In order to evaluate the influence of the status of lens (phakia/pseuodophakia) on IOP, patients were subdivided into phakic group and pseudophakic group. The changes of IOP from baseline to 24 h in phakic group and pseodophakic group were comparable (*p* = 0.422). The trend of IOP changes was similar between the two groups (Figure 2).

**FIGURE 3:**
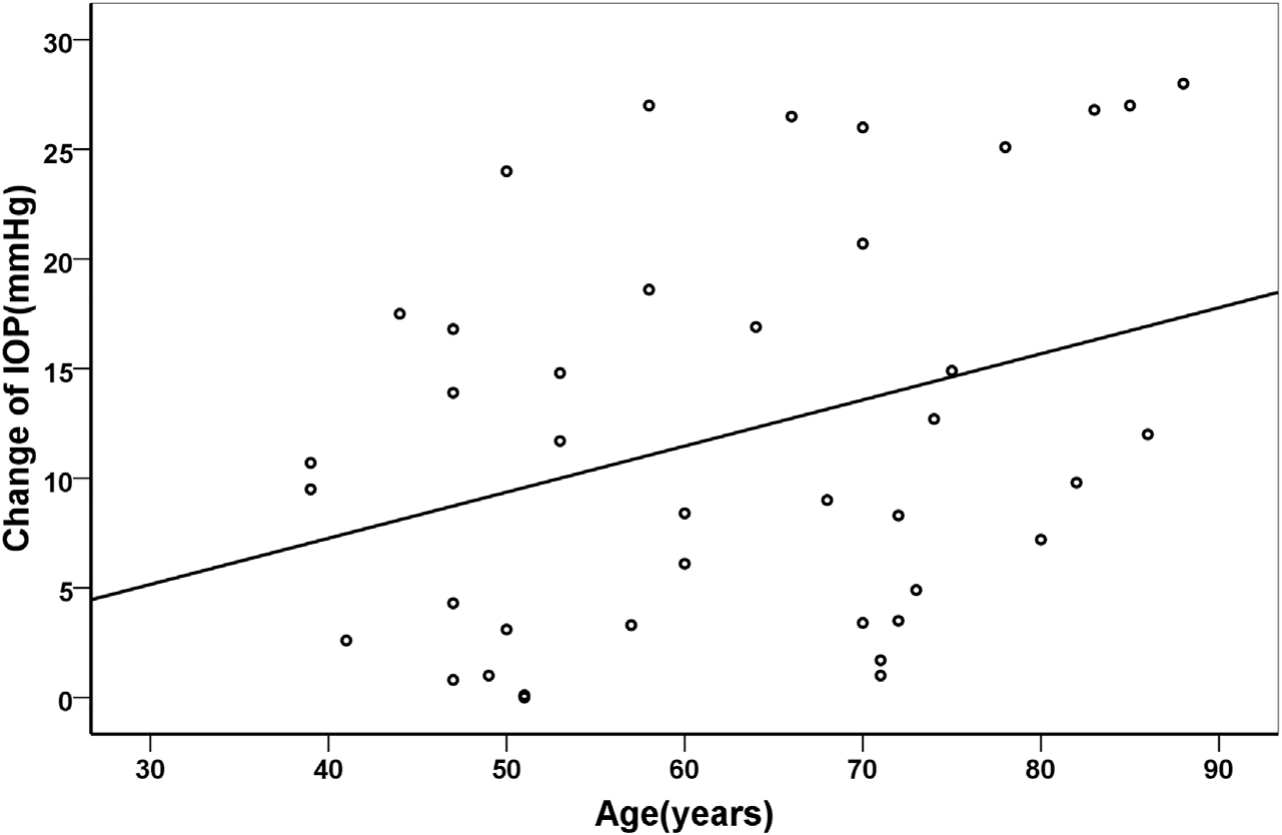
Scatter plots demonstrating the association between age and change of intraocular pressure (*r* = 0.329, *p* = 0.038).

**FIGURE 4:**
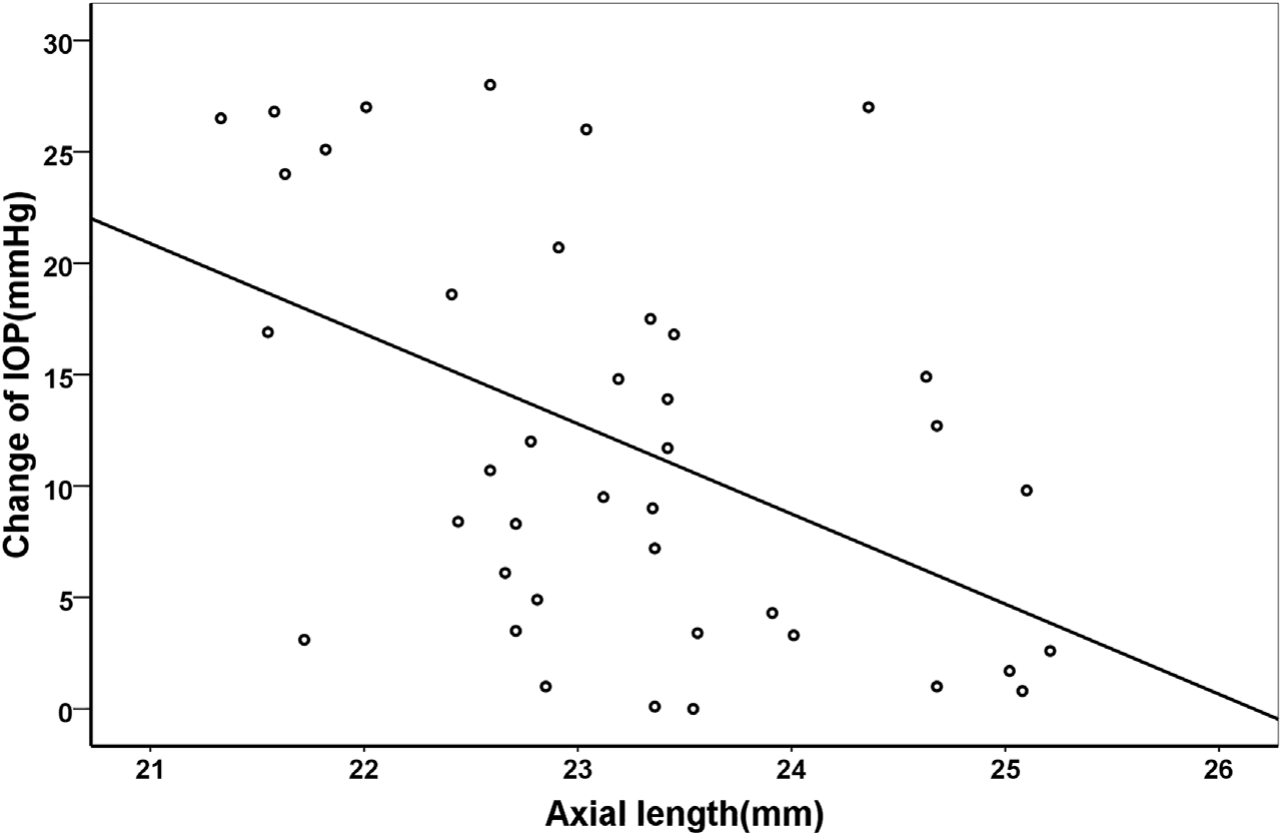
Scatter plots demonstrating the association between axial length and change of intraocular pressure (*r* = −0.472, *p* = 0.002).

Pearson correlation coefficient showed that the changes of IOP were positively correlated with age (*r* = 0.329, *p* = 0.038), but negatively with axial length (*r* = −0.472, *p* = 0.002). Their correlations were shown in Figure 3 and Figure 4, respectively.

## 4 Discussion

In the current study, we investigated the changes of IOP after the injection of Conbercept. Previous studies focused on ranibizumab, aflibercept and bevacizumab had reported similar findings (El Chehab et al., 2016; Kato et al., 2019/05; Fuest et al., 2014). To the best of our knowledge, there is only one study reporting the changes of IOP after IVI of Conbercept (Zhang et al., 2016). However, the main purpose of the study was to evaluate the influence of increased IOP on retinal nerve fiber thickness in a long-term basis. We focused on the short-term changes and monitored the IOP more accurately and closely. We found that the IOP increased dramatically after the injection and the IOP returned to baseline level after 30 min. Thus, the IOP remained high just in a short duration. Moreover, the changes of IOP were positively correlated with age, but negatively with axial length and the trend of IOP changes was similar between phakic and pseudophakic patients.

Previous studies examining the short-term changes of IOP after IVI showed that the elevated IOP returned to normal level in most patients in around 30–60 min (Mojica et al., 2008; Kato et al., 2019/05; Kim et al., 2008). We also documented similar findings in terms of IOP change after IVI of Conbercept. The pathophysiological mechanism of increased IOP after IVI is probably related to several mechanisms. The key factor affecting the IOP after IVI is the volume of anti-VEGF agents. The intraocular space is full of tissues and fluid with little room for expansion. A slight increase of volume may lead to IOP spike through the biomechanical properties of the globe. Higher injection volume with a rapid injection is associated with higher IOP spike and may potentially lead to sustained IOP elevation (Yannuzzi et al., 2014). After reviewing the reported literatures and combining with our study, we concluded that the IOP spike may not relate to the type of anti-VEGF agents, because the trend of IOP changes in IVI of ranibizumab, bevacizumab and aflibercept are similar. The transient elevation of IOP does not affect a relatively healthy eye. Lee and colleagues found that transient increase in IOP led to a transient decrease in mean ocular perfusion pressure but both parameters achieved normalization within 30 min. No ocular adverse event was found during the study follow-up period. Thus, the instant spike of IOP is generally safe without needs for medical intervention after IVI of Conbercept and paracentesis may not require in patients with DME.

Whether the status of lens affects the changes of IOP after IVI remains controversial. Demirel et al. (2015) found that pseudophakic eyes were less prone to sudden IOP spikes than phakic eyes in patients receiving ranibizumab. On the contrary, we found that no significant difference was found between the phakic group and pseudophakic group, which was consistent with previous study (El Chehab et al., 2016). In another study with larger sample size, no significant differences were observed between the phakic and pseudophakic group after IVI of 0.1 ml triamcinolone acetonide (Kerimoglu et al., 2011). However, they did found that the IOP dropped faster in pseudophakic eyes within the initial 10 min, which may result from widening of the drainage angle in pseudophakic eyes (Hayashi et al., 2000; Kerimoglu et al., 2011). Interestingly, we did not find any significant differences in terms of IOP dropping in our cohort. Nevertheless, whether the status of lens affecting the changes of IOP after IVI warrant further research.

The IOP spike after IVI is more likely to be higher in older patient. The study reporting the relationship between age and IOP changes after IVI for anti-VEGF agents is limited. The reason why IOP spikes higher in older patients may relate to the rigidity of the eyes. On one hand, previous studies have shown that the sclera may become calcified in aged subjects and the rigidity was positively correlated with age (Pallikaris et al., 2005; Grossniklaus et al., 2013). On the other hand, Sayah et al. (2021) found that the increase in IOP following IVI was more pronounced in patients with higher ocular rigidity. Thus, we assume that the expansion ability of the eye is decreased in older patients. When a certain amount of fluid is forced into the tiny vitreous cavity, the IOP spikes higher.

Apart from age, the IOP changes were also related to the axial length. Similar to the previous studies, we also found that axial length was negatively correlated to IOP changes after IVI in adults (Cacciamani et al., 2013). On the contrary, Kato et al. (2019/05) found that the IOP changes after IVI of bevacizumab in infants with retinopathy of prematurity were not associated with axial length. The controversial findings may be due to different age groups and relatively small sample size of the above study. The volume of intraocular space is relatively smaller in subjects with shorter axial length. When certain amount of anti-VEGF agent is injected into the eye, the proportion of volume increase is higher in eyes with shorter axial length. Thus, the IOP spikes could be more pronounced in patients with shorter axial length.

The current study has several limitations. The study might be more convincible if the sample size is larger. It should be noted that the injected volume was manually calibrated as all similar clinical studies. All the calibration was finished and injected by the same experienced ophthalmologist in order to ensure the precision and equal amount of anti-VEGF agent among all patients. Besides, our study did not attempt to evaluate other ocular metrics like anterior chamber depth.

## 5 Conclusion

In conclusion, our study shows that Conbercept IVI may cause a considerable transient IOP increase, and it has already been described with the three other anti-VEGF agents. Most eyes in our series achieved normalization of IOP within 30 min without needs for any immediate intervention, such as paracentesis. The IOP rise after Conbercept IVI was greater in patient with shorter axial length and older age. On the basis of these preliminary results, patients undergoing IVI of Conbercept should be carefully monitored for at least 30 min after IVI to assess IOP elevation and thus ensure adequate optic nerve head perfusion. Although IOP increase tend to normalize in most patients after 30 min, preventive paracentesis can be considered when IOP spikes after IVI are very high, especially in eyes with shorter axial lengths and older age.

## Data Availability

The raw data supporting the conclusion of this article will be made available by the authors, without undue reservation.
